# Gratuitous Medical Services

**Published:** 1863-01

**Authors:** 


### Art. VI.—GRATUITOUS MEDICAL SERVICES.

For a considerable time past, the widely prevalent custom of *giving* medical and surgical advice and assistance to the poor has been held in question by many practitioners of medicine. Being ourselves unconvinced by the arguments hitherto advanced in disfavour of the custom, we purpose to state concisely what seem to us to be the principles involved in the matter, and what are the practical results springing from their application.

The opponents to the system of gratuitous medical services appear to rest their opposition, generally speaking, upon the following grounds:—First, they deny that compliance with the custom is obligatory, as a matter of conscience and duty towards mankind; secondly, they affirm that departure from it is a duty to the profession; and thirdly, they maintain that the abuses resulting from the system are themselves sufficient to justify its unqualified condemnation.

In defence of the first statement they rest their case upon an imaginary parallel between a medical man and a baker, and the following is the argument used :—

Bread, it is alleged, is a necessary of life, in a stricter sense even than medical skill can be said to be so. The baker, in common with his fellow-citizens, lies under an obligation to supply the poor with both. But this obligation is discharged by the payment of his allotted contribution to the poor's rate. He is under no exclusive obligation to supply food gratuitously to those who cannot purchase it. He sells his bread to the authorities, who distribute it, and he gives away nothing that is special to his calling. Because the baker is not expected to give bread to all who are poor and hungry, the surgeon ought not to be expected to give skill to those who are poor and sick. Because Boards of Guardians buy bread for distribution, as much as is needed, so they, or others for them, ought to buy all the medical skill that is required, in order to meet the ailments of the poorer portion of the community.

Against some of this we have not a word to say. As a matter of fact, as far as legal obligations are concerned, the tradesman and the doctor are perfectly upon a level; and, for the service of the poor, the community is compelled to purchase what each class has to sell. The difference is this: that, while the community buys sufficient bread for all the demands, it does not buy sufficient skill, and it expects what is lacking to be made up by the medical profession. Society expects gratuitous services, not from any particular individual in the medical body, but from some of them; and this expectation is invariably fulfilled. According to our objectors, it is based upon injustice and ought not to be entertained.

To such we would reply that there is some difference (commonly, indeed, believed to be of the broadest kind), between skill and merchandize, between the gift of God and that which can be bought for money. According to Christian precept, we are bound to use our skill in healing for the benefit of the sick. Certainly, it is a gift to us, by which we are to live; but as certainly it is a gift, through us, to mankind, which, as Christian men, we dare not withhold from the poor. The baker has no gift in any like sense; and if he have a capacity for business which renders him a prosperous man, he cannot impart of this to the poor, but only of its fruits, by giving the money which it has brought him. As far, therefore, as the elementary principle is concerned, we hold all these analogies between the duty of the baker and the duty of the surgeon to be utterly fallacious. We will not urge that they are degrading, because we wish to point out a difference, not to arrogate a superiority.

If this view be correct, the first objection advanced falls to the ground, and with it, subject to a higher law of duty, the second. But it would be well to reflect whether there is not another aspect than that advocated by the objectors, in which duty to the profession may be regarded. It will hardly be denied that the power of healing is a personal gift, not possessed by all people in an equal degree, and which the mere compliance with the requirements of a professional curriculum and of boards of examiners can hardly supply. It is a gift, indeed, to be cultivated, and increased by study and observation, but scarcely, we think, to be altogether acquired. But there is a tacit assumption, underlying the outcry against which we protest, that all qualified practitioners are equal in skill and knowledge. No one has absolutely put this assumption into words, but the idea is never absent from the discussion. The hospital surgeon, it is said, who sees a labourer without a fee, deprives the general practitioner of a certain modicum of profit, it matters not how small. The hospital surgeon cannot take less than a guinea, the general practitioner may fittingly receive one-eighth of that sum, for which he will gladly supply, not only advice, but also physic. The labourer cannot pay the larger fee, but he might probably pay the smaller. The hospital surgeon, therefore, by his compliance with an evil custom, robs the general practitioner of his modest emolument, and diminishes, by so much, the aggregate earnings of the profession during the day. To give force to this argument, however, it is necessary to assume that the patient would derive equal benefit whether treated by the general practitioner or the hospital surgeon. It is only, in fact, upon this assumption that the argument can have any weight.

We willingly concede that hospital staffs do not hold sole possession either of the gift of healing or of medical wisdom. Happily this is very far from being the case ; but it is well to consider whether hospital physicians and surgeons do not perform a somewhat higher duty towards the profession than that which may be estimated by pounds, shillings, and pence ; whether, in fact, their gratuitous services are not only a work of Christian charity, but also a reasonable tribute to the public, on behalf of the profession, for those errors and shortcomings necessary to a science imperfect in itself and often most imperfectly practised. Does not the profession, in fact, show a higher appreciation of its lofty calling, and perform more nobly its duty to itself, by offering, untarnished by greed, a ready succour, not only to the utterly impoverished, but to the victims of erring, careless, ignorant, or incompetent brethren? That there are such, few, we presume, will deny. How, indeed, could it be otherwise, when we reflect upon the very inadequate preparation which has hitherto been required, either professionally or morally, for the responsibilities of a medical life? Our examining bodies have framed regulations, which chiefly secure fees for themselves, a period of empirical drudgery or of chartered idleness for the student, and a few weeks of cramming by a grinder, as a preparation for the final ordeal. It is true that they have lately been forced, by public opinion, into reforms, but they are even now looking back with regret upon the good old times of idleness and ignorance, and they have reformed as little as they dared. There can be no higher or better testimony to the essential tendency of medical practice to elevate those who pursue it, than the fact that, in spite of the College of Surgeons and the Apothecaries' Hall, in spite of apprenticeship and grinding, the great bulk of the general practitioners of this country do actually struggle out of the slough of student life into the position of honourable and intelligent gentlemen. But there are many who do not do this; and it is to be remembered that it is upon these that the paid treatment of the poor must chiefly devolve. For various reasons such men do not "get on," and they are compelled to work for a remuneration which more prosperous men refuse. They are compelled to work, but they cannot be compelled to work well.

We are painfully impressed by the conviction (forced upon us by long and wide experience), that the medical treatment of the sick poor, whether as club or union patients, or as paying small sums for medicine upon its delivery, is often unsatisfactory in the last degree. "Cheapness," is the great object sought under these circumstances, and the medical care is too commonly accurately apportioned to the amount paid per case. If a man's necessity unfortunately compel him to accept the paltry terms offered by a "union" or a "club," his necessities, it is well to remember, do not compel him to treat his pauper or club patients indifferently or carelessly. The moral responsibility in these cases differs not a whit from that of better paid cases; the responsibility, in fact, cannot be measured by the scale of charges. The moral guilt of a board of guardians or club does not cover moral guilt on the part of the parochial or club medical officer. But often necessity has nothing whatever to do with the acceptance of the beggarly pittances proffered by these bodies. The following recent example is a case in point:—

The poor of a township under the "Gilbert Act," had been long attended by a medical practitioner, whose charges, including medicine, averaged about 30*l.* a year. On a change of guardians the board was seized with an economical fit, and it determined that the medical cost of the poor should, in future, as in neighbouring townships, be arranged by estimate "in lump," and not, as previously, by charge per case. Notices were distributed to the three medical practitioners residing within the township, asking them to send in estimates of the sum for which they would each undertake the medical care of the poor. No information could be given as to the average amount of sick in the year, records of medical relief solely not being kept. One practitioner refused to give an estimate; the other two (one having been for some time the township medical officer) sent in estimates for 30*l.*, stating that they named this sum because it approximated to the annual average and reasonable medical cost of several years. In the meantime, the project of the guardians had become widely known, and estimates poured in from surgeons in adjoining townships. All these practitioners lived *out* of the township, the said township being somewhat extensive and containing not less than 8500 population. The lowest of the volunteered estimates was 5*l.*, the highest 20*l.* The practitioner who offered to do for 20*l.* the work required, lived a mile and three quarters from the workhouse, and near to one extremity of the township, but about two miles and a half from the furthest extremity. The practitioner who offered to do the work for 5*l.*, lived upwards of two miles distant from any part of the township. A third practitioner, who gave an estimate of 10*l.*, lived a mile and a half distant from the nearest cottage in the township. The two last-named individuals were comparatively substantial men. They both stated that they had private patients in the township, and that as they worked their practice entirely riding or driving, the distance would in no wise affect their proper attendance upon the sick. As to the facts that the distance of their residences would in any way tell somewhat heavily upon the pauper invalid who had to seek their help, and would interpose a somewhat serious difficulty to obtaining immediate assistance in sudden illness, or quick attendance in midwifery cases; or that the loss of half a day to some member of the sick person's family, in fetching the medicine (the estimate including the supply of medicine!)—these of course were trifling questions which such worthy gentlemen could not be expected to entertain. The guardians long debated the propriety of electing the gentleman referred to whose estimate amounted to 10*l.* One great argument in his favour was that he had attended the poor of his own township, the population of which was about 7000, for a like sum for several years. It was feared, however, that as the practitioner lived out of the township the election would be illegal. In the end, the resident practitioners, who had given estimates, refusing to modify their estimates, the practitioner who had offered to do the work for 20*l.* was elected, he (a new-comer) living nearest to the township. We had the curiosity to ask this brother-practitioner upon what principle he had given his estimate. "Oh," he remarked, jauntily, "the amount is my house-rent, and I'll make it pay, you'll see." "And how about the sick poor," we inquired. "That is their look-out," he laughingly rejoined, "and the look-out of the Board of Guardians." We have a certain weakness for the credit and honour of the profession, which we are sometimes at a loss whether to look upon as a virtue or as a vice. It is not easy at all times to determine whether reticence belongs to the one category or the other. The worthy we have referred to is a "M.R.C.S. and L.A.C." of no very late date, and he was accustomed to describe us as "A very good fellow, always ready to get a brother practitioner out of a *mess*." Within a fortnight we were summoned hastily to aid this representative of medicine in the case of an aged pauper. He (i.e. the surgeon) had contrived to thrust a catheter through the wall of the urethra, and alongside the rectum, and he was puzzled that urine did not follow. A day or two afterwards he met with an instance of what he called "impaction" in a pauper midwifery case, and he opened the child's head with a pair of ordinary, short scissors, with "immediate success." As for the more serious surgical cases he came across, it was merely from the fact that he had a neighbouring general hospital to fall back upon, that the unfortunates were saved from lamentable results. Thanks to our kindness and the general hospital, the man, for the sake of his large family, was saved from immediate perdition, but he was quickly deprived of doing mischief upon the sick poor; and it is to be hoped that wherever he may now be neither poor nor rich may be submitted to his ignorant mercies.

It may be said that this is a rare case. It is a glaring case, we admit, but it is not a solitary case within our own experience, and it is but one of many in which the profession has escaped unutterable disgrace almost solely through the generous, gratuitous, and oft self-sacrificing aid which it affords to the poor, privately and in hospitals. We have a right to place this aid as a set-off against the short-comings of ignorance and error, and to glory in the set-off.

But there is another consideration, of wider application, perhaps, and which leads to very nigh the same conclusion.

As long as medical science is progressive, it must always be extending its boundaries in some direction or other, more rapidly than busy men in general practice can keep pace with it. Of such partial developments, such processes of outgrowth from the general body of knowledge, several have been witnessed by the generation now living. When the out-patients of the London Hospital described Dr. Thomas Davies as "the man with the horn," auscultation of the lungs was a mystery to the majority of the profession. Since then auscultation of the heart, and a knowledge of the true significance of its sounds, and more lately still certain facts about the pathology of cerebral and spinal diseases, have been of practical utility in the hands of a few, long before they could be mastered and applied by the many. During the last few years, the marvellous advances of ophthalmology furnish another and very striking illustration of the same kind; while the recent improvements in laryngoscopy and rhinoscopy are only less striking because more restricted in their application. We acquiesce contentedly in the fact that, perhaps not one practitioner in twenty can use the ophthalmoscope and correctly interpret its revelations, although it has now been more than ten years before the profession, and although no one, without faithlessness both to the profession and patient, can attempt to treat the internal diseases of the eye without it. Surely our acquiescence depends upon the effect of custom, in shutting the eyes to the most glaring evils. If twenty men all alike profess to treat disease, and nineteen of them will not take the trouble to acquire a certain amount of tact and knowledge, without which the treatment of a very important class of diseases cannot be conducted, do not the nineteen commit a most serious breach of a sacred duty? Is it not obligatory upon them that they should be armed at all points, and prepared to deserve the confidence they solicit? And if, as often happens, when one of the nineteen men is applied to by a patient with internal eye disease, by a poor man, let us say, instead of confessing his ignorance on the matter, and referring the sufferer to his better instructed brother, he treats him without knowledge and without benefit, gives him medicines that may be useless, or even hurtful, and pockets his money, does he not commit a heartless wrong? How can he complain if the twentieth man gives his skill freely to the poor? Is it not to the profit and credit of the profession, as a whole, that the patient should be restored to sight *gratis* by A., rather than that he should be confirmed in blindness by paying half-crowns to B.? Is it not to the credit of the profession that A. should remove all shadow of justification from B.'s misconduct, and deprive the poor, so far as A.'s special knowledge will go, of any reason for seeking B. on account of his presumed cheapness?

We have taken an illustration from ophthalmology because, in its present condition, it furnishes very striking ones. Mr. Bowman has lately published these remarkable words:—

"These (glaucomatous) diseases now begin to admit of accurate definition, of exact discrimination, and, in many cases, of the most admirable cures—diseases which, six years ago, marched on unchecked to more or less rapid destruction of sight. And I do not scruple to say that, were the scientific knowledge of them, now possessed by a few, diffused universally among all the members of our profession, failure of sight from this cause, in Great Britain, would be to a considerable degree prevented, and total blindness would be rare indeed." *

In the face of these words it cannot be disputed that, until all practitioners acquire the knowledge Mr. Bowman indicates, he and his colleagues support the dignity of the profession, while they render vast services to the community.

So long, then, as careless and ignorant practitioners are to be found ; so long as a few allow the progress of science to pass on and leave them behind ; so long, we conceive, will it be a noble work to afford a refuge to the poor, where, without being mulcted in purse, they can be secured in very great measure against both the errors of haste and the errors of ignorance. Both reflect infinite discredit upon the profession ; and it is fit that we should, as a body, provide gratuitously the remedy for our own shortcomings.

With regard to the presumed "abuses" of the gratuitous advice system, it is certain, we think, that no system of human contrivance does the same amount of good with so little evil to counterbalance it. Those who object to the gratuitous character of medical services to charitable institutions, would not, we presume, propose to shut up the institutions altogether, but only to remunerate the medical officers. Let us see what would be the practical results of such a proceeding.

In the first place, a demand for payment by medical officers, would at once close every struggling institution, and every institution of small size and limited income. If payment be given, it must, of course, be a sum bearing proper relation to the professional station of the receiver, and to the time devoted to the work. We are acquainted with one dispensary, where the surgeons receive 10*l.* per annum each, as an acknowledgment of their right to payment, and, in fact, as the small end of the wedge. This ground for receiving a housemaid's wages is all very well, as long as such a payment is exceptional, and avowedly made to assert a principle. But if the rule were for medical officers to receive a pecuniary recompense, that recompense must be a real remuneration for their skill, and must harmonize with their ordinary scale of fees. It will be easy for

* *British Medical Journal*, Oct. 11th, 1862.

any reader to fix in his own mind upon an adequate sum; to multiply it by the number of the medical staff of the nearest county hospital; and then to consider whence the money is to be obtained. Generally speaking, hospitals and dispensaries have not funds enough to encounter the sickness and accidents of their districts. Their normal condition is one of indebtedness to their treasurers or bankers; and their ordinary resource is some form of "special appeal." In point of fact, their existence is possible only because their physicians and surgeons work for nothing.

It cannot be disputed, perhaps, that the position of an honorary officer must always be more dignified than that of a stipendiary. The honorary surgeon, or physician, meets the gentlemen who compose the weekly board on a footing of perfect equality—as their fellow-labourer in a charitable work. The stipendiary would meet them in the hospital only as their paid servant; and would, in many ways, be liable to a control that might easily be made vexatious. It would be no slight evil, surely, to degrade the leading members of the medical profession in the relation they hold to the most influential and wealthy of their patients.

It would be difficult, moreover, to fix upon any scale of payment that would not lead to the suggestion, in times of poverty or pressure, that the work might be done for less. When a hospital was in difficulties, the chairman would say in confidence to the senior surgeon (who was paid, let us say, 400*l.* a-year), that Mr. So-and-so would be very glad to take the office for 200*l.*; and that the institution ought not to lose the opportunity of effecting so important a saving under the head of "salaries." We fully believe that if every hospital and dispensary in the kingdom were to commence the new year with a salaried staff, the application of this kind of argument would restore the "gratis system" before the lapse of a single generation.

Among the so-called "abuses" of our hospital system, the one that is most chiefly and continuously urged is the relief afforded to persons "able to pay." We do not see how stipendiary officers would remove this evil, unless by affording to grumblers the satisfaction of knowing that somebody was paid. In order that this satisfaction should be complete, the contemplated remuneration should be "per case."

It is quite plain, we think, that if persons in easy circumstances choose to be dishonest, and to resort to certain subterfuges, they may obtain the advantages of charitable institutions with very little risk of detection. It is not, and in the nature of things it cannot be, anybody's business to find them out.

They can only be excluded by closing the doors altogether, and most persons would consider the remedy worse than the evil.

There are many cases, however, where an apparent ability to pay is a merely deceptive appearance; where those who have had losses, or been reduced by some of the various kinds of misfortune, are struggling to maintain a decent appearance, or to keep their footing in their class of life until better times may come. We have followed up the history of a few of the seemingly "better-off" hospital patients, and, in doing so have been brought face to face with some of the saddest stories of privation ever encountered in the course of a professional career. For such people, gratuitous medical service means the detection of the germs of disease, the treatment of ailments for which the sufferers would not have afforded themselves a paid doctor, and thus, over and over again, the salvation of lives valuable to families and to the community.

But, it is averred, farmer Smith and butcher Brown go to the county infirmary as out-patients, or go by excursion train to London, and get advice at a hospital there. We do not believe it. Their wives possibly may do so; but attendance at a hospital absorbs too much time to be practised by any class whose time is not absolutely worthless. And to whatever extent the objection may be true, its truth, as a rule, is a mere expression of the popular feeling that doctors of only very moderate repute are more careful to lay the foundation of a bill of charges than to investigate and cure the diseases brought under their notice.

We have our own complaint against hospitals, and it is of a very serious character. They do not accomplish nearly what their pecuniary resources would allow, on account of the most unwise, and, indeed, unjust limitation of the number of their medical officers. In nearly every county town the hospital is in the hands of a small and exclusive clique, who, by virtue of their appointments, affect superiority to their brethren. These appointments are obtained by election, after a costly and humiliating canvass, often after a contest in which every kind of electioneering trick is practised, and in which family interest, religious creed, or other considerations wholly foreign to the issue, are of equal, sometimes of far more, weight than the possession of professional skill, and the conscientious discharge of professional duty. The number of the staff thus selected is invariably below the real needs of the institution, with the result that the out-patient department is grievously neglected, and often handed over absolutely to the house-surgeon. The needs of the institution should, we think, be even less the measure of the proper strength of the staff, than the number of willing labourers that could be procured. The profession, as a body, supports hospitals nobly.

The profession, as a body, has a right to all the advantages (of which experience is the chief) to be gained from them. We hold that every physician or surgeon who resides within a certain distance of a hospital, and who gives proof of skill and diligence in his calling, ought to be permitted to take his share of the work. The duties of a hospital might in this way be divided among a sufficient number of men to insure their proper performance; the profession would largely benefit by the wide distribution of the privileges now so jealously guarded by a few; the patients, by the greater amount of time and care bestowed upon them; and the public, by the power of selection among many men, to each of whom the advantages of hospital practice and of hospital responsibility had been accorded.

